# Genetic evidence of extra-pair paternity and intraspecific brood parasitism in the monk parakeet

**DOI:** 10.1186/1742-9994-10-68

**Published:** 2013-11-09

**Authors:** Juan José Martínez, María Carla de Aranzamendi, Juan F Masello, Enrique H Bucher

**Affiliations:** 1 Facultad de Ciencias Exactas, Físicas y Naturales, Universidad Nacional de Córdoba, Av. Vélez Sarsfield 299, (5000) Córdoba, Argentina; 2 Ecología Marina. Instituto de Diversidad y Ecología Animal (CONICET-UNC) and Facultad de Ciencias Exactas, Físicas y Naturales, Universidad Nacional de Córdoba, Avenida Vélez Sarsfield 299, (5000) Córdoba, Argentina; 3 Max Planck Institute for Ornithology, Vogelwarte Radolfzell, Radolfzell, Germany; 4 Centro de Zoología Aplicada. Instituto de Diversidad y Ecología Animal (CONICET-UNC) and Facultad de Ciencias Exactas, Físicas y Naturales, Universidad Nacional de Córdoba, Avenida Vélez Sarsfield 299, (5000) Córdoba, Argentina; 5 Present address: Departamento de Ciencias Naturales, Facultad de Ciencias Exactas, Físico-Químicas y Naturales, Consejo Nacional de Investigaciones Científicas y Técnicas (CONICET), Universidad Nacional de Río Cuarto, Ruta Nacional 36 Km 601, Río Cuarto 5800 Argentina

**Keywords:** Parrots, Extra-pair paternity, Intra-brood parasitism, Breeding biology, Argentina

## Abstract

**Introduction:**

The monk parakeet (*Myiopsitta monachus*) is a widespread invasive species native to southern South America that has become established in many regions of the world. Monk parakeets breed in a large, fully enclosed structure built from twigs, which consist of one to many individual brooding chambers. The species has been considered to be socially and genetically monogamous. However, genetic relatedness of adults to juveniles in the native area was found to be lower than expected for monogamy. To assess the significance of this discrepancy, we examined individual and population genetic patterns of microsatellite loci at two sites in Córdoba province, Argentina.

**Results:**

We sampled 154 nestlings and 42 adults in Córdoba, Argentina. Mean value of pairwise relatedness of nestlings within chambers was about 0.40. Contrarily, relatedness of nestlings between chambers was close to zero. We found a considerable degree of variation in nestling pairwise relatedness and parentage within chambers, including chambers with combinations of unrelated, half-sib, and full-sib nestlings. The proportion of sibling relatedness indicated monogamy in 47% and extra pair-paternity in 40% of the chambers. We also found intra-brood parasitism in 3% of the chambers.

**Conclusions:**

Our results indicate that the monk parakeet is sexually polygamous in its native range in Argentina, which is consistent with the observed mean value of relatedness of adults to juveniles of about 0.4. We also confirm the existence of intra-brood parasitism. High density of monk parakeets may favor occurrence of extra-pair paternity and intra-brood parasitism in the native sites.

## Introduction

The monk parakeet is a South American species unique among parrots because its communal nests allow independence from tree or cliff cavities as the required breeding habitat by most parrots. Nesting habitat flexibility may contribute substantially to the considerable success of the monk parakeet as an invasive species, having already expanded in several countries in South, Central and North America, as well as in Europe, the Caribbean and Japan [1]. In addition, in many regions the monk parakeet is considered a problem for agriculture and also for electricity transmission lines [2]. Accordingly, the monk parakeet has attracted considerable attention and research effort, not only because of practical management needs, but also because of its unique ecological and behavioural characteristics [3].

Monk parakeets nest in a large, fully enclosed bulky structure, built from twigs on the topmost branches of a tree. They build the nest by adding individual brooding chambers, each with its own entrance tunnel and no connection between compartments, where they lay the eggs [4]. Accordingly, nests may include from a single chamber up to over 200 chambers, although in natural settings nests typically include l-4 chambers [3,5]. Nests are used not only for breeding, but also as roosting quarters by breeding and non-breeding monk parakeets throughout the year, and they always roost inside a nest.

The monk parakeet has been reported to be socially and genetically monogamous [4-6]. However, breeding attempts including trios have been reported occasionally [5,7]. In recent years, a study on population genetics has provided valuable information on the breeding behaviour of the monk parakeet. Gonçalves da Silva *et al.*[6] found strong evidence for sexual monogamy from patterns of relatedness within sites in native monk parakeet populations of Argentina (60 individuals) and invasive populations of the United States (195 individuals). Secondly, the authors found no definite cases of extra-pair paternity in either population.

However, Gonçalves da Silva *et al.*[6] also found that mean relatedness of both adult females and males to juveniles in Argentina was significantly lower than the expected genetic relatedness value (0.5) for monogamy. In addition, they also found one potential instance of extra-pair paternity in Argentina, which was disregarded due to limited information available at the moment of conducting their research. Accordingly, and in view of the low relatedness value found in Argentina, Gonçalves da Silva *et al.*[6] suggested a wider geographical sampling to ascertain the biological significance of the observed difference between Argentina and USA populations.

Clearly, this open question is of fundamental importance in terms of the understanding of the monk parakeet breeding behaviour, and also in the context of possible behavioural differences between native and invasive sites that may have emerged in the invasive sites [6]. Here we present an analysis of relatedness, sibship reconstruction and extra-pair paternity analysis in monk parakeet nests from two 20-km distant sites in central Argentina (i.e., Marull and Miramar, Córdoba province), using a microsatellite-based genotyping method. Specifically, we investigated: (1) the relatedness between nestlings in each breeding chamber in single and multi-chambered nests; (2) the relatedness between nestlings from different chambers within the same compound nest; (3) the paternity and maternity of adults captured in nests. Special attention was given to the possible existence of extra-pair paternity and intra-brood brood parasitism.

## Results

We sampled 28 nests with 37 chambers distributed as follows: 21 chambers in single-chamber nests, 12 in six two-chamber nests, and four in one four-chamber nest. We captured and blood sampled 154 nestlings and 42 adults. Of the adults, 21 were males and 20 females (one individual was not able to be sexed due to the failure in amplify the sex-linked molecular marker). Nestling-adult relationship was found in 18 adults.

Of the remaining 24 adults with no parentage relationship, 18 were trapped in the studied nests (listed in Additional file 1), whereas six additional individuals were captured in the Miramar area in nests not included in the nestling sampling.

### Genetic variation

The analyses to detect the presence of null alleles were significant in five of seven loci (AgGT019, AgGT029, AgGT090, MmGT054 and MmGT057). However, only the loci AgGT090 and MmGT054 deviated significantly from Hardy-Weinberg (H-W) equilibrium when the three data sets were taken into account (i.e., adults, juveniles and, adults and juveniles together) (data not shown). Even though those loci presented evidence for the occurrence of null alleles, we included these loci in further analyses due to their high polymorphism, following Wagner *et al.*[8] (Additional file 2).

The results of AMOVA are shown in Table 1. Genetic variation was explained by within-individuals partition (78%), whereas differences among individuals within localities explained 21% of the genetic variation observed. The between-sites partition was not significant.

**Table 1:** Summary of Analysis of Molecular Variance (AMOVA) among adult individuals from Marull and Miramar monk parakeet populations

**(A) Source of variation**	**Sum of squares**	**Variance components**	**Percentage of variation**
-Between sites (Marull and Miramar)	4.104	0.024	0.917
-Among individuals within sites	123.384	0.554	21.069
-Within individuals	84.500	2.051	78.015
-Total	211.988	2.629	
(B) Fixation index	Estimated value (*P*)		
*F*_*is*_	0.213 (< 0.001)		
*F*_*st*_	0.009 (0.801)		
*F*_*it*_	0.219 (< 0.001)		

### Patterns of relatedness in nests

#### Nestlings

**Figure 1:**
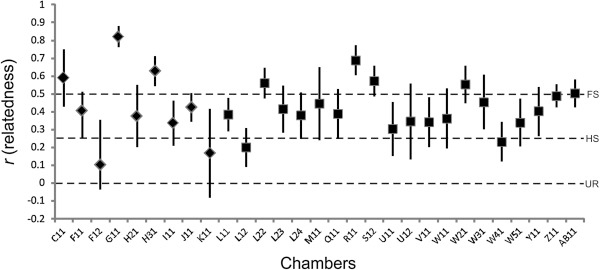
**Mean pairwise relatedness values (r) for nestlings within chambers.** Dotted lines indicate expected values of relatedness for UR: unrelated (r = 0), HS: half-sibs (r = 0.25) and FS: full-sibs (r = 0.5). Diamonds: chambers from Marull; Squares: chambers from Miramar.

Mean value of pairwise relatedness of nestlings within chambers (including chambers in compound nests) was about 0.41 for both localities, a lower value than that expected under sexual monogamy (0.50) (Table 2) and consistent with the previous study. Figure 1 depicts the variation of pairwise relatedness within chambers with more than three nestlings. Contrarily, mean value of pairwise relatedness of nestlings between chambers was zero in Miramar and close to zero in Marull. For cases of nests with two or more chambers, the mean pairwise relatedness among juveniles was very low, zero in Miramar and close to zero in Marull.

**Table 2:** Population structure of pairwise relatedness values

**Pairwise comparisons**	**Expected value under sexual monogamy**	**Location**			
		***Marull***		***Miramar***	
		**Comparisons N**	**Mean (95% CI)**	**Comparisons N**	**Mean (95% CI)**
Individuals within chambers	0.5	80	0.418 (0.355–0.482)	215	0.415 (0.381–0.448)
Individuals among chambers	0.0	866	0.034 (0.019–0.049)	5780	−0.049 (−0.055– -0.044)
Individuals within nests	0.0	34	0.028 (−0.050–0.105)	174	−0.013 (−0.045–0.019)
Adult females to juveniles within nests	0.5	50	0.162 (0.085–0.238)	107	0.229 (0.178–0.279)
Adult females to juveniles among nests	0.0	302	−0.053 (−0.076– -0.029)	1213	−0.035 (−0.047– -0.023)
Adult males to juveniles within nests	0.5	44	0.214 (0.127- 0.301)	114	0.171 (0118–0.224)
Adult males to juveniles among nests	0.0	264	0.041 (0.013–0.068)	1426	−0.018 (−0.029– - 0.007)

#### Adults

Comparison of adult to juvenile relatedness within and among nests indicates some slight differences between localities. Mean pairwise relatedness between candidate mothers and juveniles was low in relation to the expected value for sexual monogamy (0.5). Observed mean value in Miramar was slightly higher than in Marull. Mean pairwise relatedness between candidate fathers and juveniles showed values similar to those found in females, although in Miramar the mean relatedness value was lower than in Marull (Table 2).

### Variations in genetic relatedness among nests and chambers

We found a considerable degree of variation in nestling pairwise relatedness and parentage within chambers, including chambers with combinations of unrelated, half-sibs, and full-sibs nestlings (Additional file 1). Of the 37 chambers with nestlings, the proportion of different degrees of sibling relatedness was as follows: 18 (47%) had full-sibs, with two of them also having one (in one case) or two (in two cases) unrelated individuals; 15 (40%) had combinations of half- and full-sibs, and one (3%) had one unrelated individual. We also found genetic parents in different nests or chambers from their nestling location (Additional file 1).

In the remaining four chambers (10%) we were not able to determine relatedness with acceptable probability (three cases with a single nestling and one case with five nestlings and three adults) (Additional file 1).

With regards to adults captured in nests, in five of the nine monogamy chambers with adults we found the genetic pair with their nestlings in the breeding chamber. In two other chambers, only the genetic mother was present, together with one and three unrelated adults, respectively. In the remaining three chambers we found unrelated adults only. We found adults genetically related to the nestlings in four of the seven polygamy chambers. In one case the two genetic parents (of a subgroup of three half-sib nestlings in a clutch of five) were in the breeding chamber. In three other chambers we trapped only one genetic parent. The remaining three chambers had unrelated individuals only (Additional file 1).

## Discussion

Our study confirms that values below the 0.5 threshold in monk parakeet adult-juvenile relatedness are widespread in Argentina, adding to a similar value found by Gonçalves *et al.*[6] in a population sampled in Entre Rios province (about 400 km east from our sites in Cordoba).

The observed low relatedness values are clearly consistent with occurrence of a significant rate of extra-pair paternity in about half of the sampled breeding chambers, together with cases of intra-brood parasitism.

Further evidence is provided by the three detected cases of adult males with nestlings in two different nests. A potential case of extra-pair paternity in the monk parakeet was first mentioned by Gonçalves *et al.* in Entre Rios, Argentina [6], which was disregarded, although the authors mentioned that their approach might be too conservative.

Our finding of extra-pair paternity in monk parakeet drastically changes the until now generalized concept of almost absolute prevalence of monogamy among parrots [9], since no conclusive evidence of extra-pair paternity has been found previously, including a detailed study of the colonial breeding burrowing parrot (*Cyanoliseus patagonus*) [10]. It is likely, however, that further research may find new cases of polygamy in the Psittacidae, given that since application of molecular techniques extra-pair paternity has been found to occur widely in birds [11]. For example, there is preliminary evidence of extra-pair paternity in the African echo parakeet (*Psittacula eques*) [12], and in the blue-and-yellow macaw (*Ara ararauna*) [13].

Variations in intraspecific reproductive strategies between native and invasive sites may result from an adaptive response to environmental changes or population density [14]. Although inconclusive, there is good evidence that breeding density may be important in determining variation in the extra-pair paternity rate at the species level, as shown by Møller & Ninni [15] and Westneat & Sherman [16]. In this regard, our two localities (Marull and Miramar) are located in an area of high monk parakeet density in the province of Córdoba [17], whereas it is likely that populations in invasive sites are still growing, and therefore less dense than areas that sustain long-time occupation as in Córdoba. Differences in density may explain therefore the higher relatedness values found in the USA by Gonçalves *et al.*[6].

Existence of intra-brood parasitism in the monk parakeet is based on the finding of three chambers with four nestlings unrelated to the rest of the brood (3% of the 154 nestlings sampled). The three cases correspond to nests with more than one chamber, suggesting that intra-brood parasitism may be related to multi-chambered nests. This possibility is further supported by the capture of the mother of one unrelated nestling in the neighbour chamber of the same nest (Additional file 1).

Additional evidence of intra-brood parasitism in monk parakeets is provided by the finding of supernumerary clutches of about twice the average number [18,19]. In a detailed study of a monk parakeet nest [18], the author found a single chamber with supernumerary clutches for two consecutive years (12 and 11 eggs, respectively). Eggs were initially laid every second day, but after a while, new eggs appeared daily (one or two). Later, egg-laying returned to the initial rhythm until the end of the period. According to Yom-Tov [20], irregular sequence of appearance of eggs and abnormally large clutches of about twice the normal size suggest that the extra eggs were laid by more than one female.

Occurrence of intra-brood parasitism has also been recorded in the burrowing parrot, with two cases of unrelated nestlings found in a sample of 166 nestlings (1.2%) [10]. One of these cases may have resulted from brood mixing from a neighbouring nest with a collapsed wall. However, the second case could not be explained by brood mixing, and therefore most likely resulted from intraspecific brood parasitism. Even if brood mixing cannot be discarded in monk parakeet nests, in our case this possibility is unlikely, given that all collected nestlings were very young and therefore unable to displace by themselves. The probable occurrence of intra-brood parasitism was also reported in the green-rumped parrotlet (*Forpus passerinus*) by Beissinger & Waltman [21], based on the existence of larger than average clutches.

High population density may also favor intra-brood parasitism. Supporting evidence indicating that in some bird populations high population density was correlated with nest parasitism was reviewed by Spoon [9] and Griffith *et al.*[11]. Another possible factor favouring intra-brood parasitism is colonial breeding, and particularly the close proximity of nests (chambers) in the monk parakeet communal nests. Rohwer & Freeman [22] found evidence of greater conspecific nest parasitism in those parentally fed species that nest in colonies, as compared with species with dispersed nests.

It is also likely that occurrence of extra-pair paternity and intra-brood parasitism may be favoured in areas of high monk parakeet density where nests have a larger number of breeding chambers in the same compound nests, and also nests tend to group in close proximity either in the same or neighbouring trees forming the so called “colonies” [5], in contrast with invasive sites with lower density and smaller size of the compound nests. Unfortunately, understanding the adaptive significance of both extra-pair paternity and intra-brood parasitism in the monk parakeet is constrained by the limited information available on the species’ life story, hindering a more comprehensive analysis of the interaction and conflicts of life-history traits [23].

## Conclusions

Our results confirm that in the monk parakeet native range in Argentina mean values of relatedness of adults to juveniles are lower than expected for monogamy. We also found evidence of important levels of extra-pair paternity (40% of chambers) and four cases of intra-brood parasitism in 3% of chambers.

## Materials and methods

### Sampling sites and field methods

Samples were collected from two sites located 20 km apart in central Argentina (Córdoba province): Marull (31º 40' S, 62º 49 W) and Miramar (32º 55' S, 62º 40' W). Both localities are situated in the Pampas ecoregion, a vast plain landscape that is almost entirely used for agriculture. Nests were examined during the last week of November and the first week of December 2000. During that period, all breeding chambers contained only young, flightless nestlings, since the monk parakeet has a highly synchronized, single annual brood [19].

Nests were located in eucalyptus tree rows along fences, over 15 m above ground level. We reached the nests during the night using a cherry picker truck. Individuals found in each chamber (nestlings and adults) were captured with small funnel nets (placed at the opening of each breeding chamber). In both sites trapping took place immediately after arrival of researchers at dusk, in order to minimize disturbance. We did not capture all adult individuals in nests, because some escaped when we were approaching the nets. We are almost certain however that all of the trapped individuals were roosting in the chambers where they were captured, as we did not find openings between chambers that could allow adults to move at the moment of trapping. We did not observe or mark the adult population in the area before the trapping day. Therefore, no information is available on the social status of the trapped adults besides their chamber location.

Individuals were blood-sampled, banded, and kept in cages. Early in the following morning nestlings were returned to the nest and adults were released near the capture site. A total of 28 nests were sampled (Miramar: 19, Marull: 9): 21 nests had a single chamber, six nests had two chambers, and one nest had four chambers. Average number of nestlings per chamber was 4.6 (SD = 1.6, ranging between one and eight). A total of 196 individuals were genotyped (154 nestlings, and 42 adults, including 21 candidate fathers, 20 candidate mothers and one adult individual that we were unable to sex).

### DNA extraction and PCR amplifications

Genomic DNA was extracted from blood samples according to the protocol 1 of salt extraction [24]. Seven loci of microsatellites were used for relatedness and parentage analyses: AgGT19, AgGT29, AgGT90 [25], MmGT046, MmGT054, MmGT057 and MmGT060 [26]. PCR reactions were performed in a Master cycler Eppendorf® (Hamburg, Germany) in 10 μl volume containing: ca. 10 ng of DNA, 75 mM Tris–HCl pH 8.8, 20 mM (NH4)SO4, 0.01% Tween-20, 1 mM of MgCl2 (1.5 mM of MgCl2 for AgGT90), 62.5 mM of dNTPs, 20 μM of each primer and 0.5 U of Taq DNA polymerase (Fermentas, Brazil). Reaction conditions were the following: initial step at 95°C for 10 min, 30 cycles at 95°C for 30 s, 57°C for 30 s, 72°C for 30 s and a final extension at 72°C for 10 min. For AgGT29, MmGT046, MmGT054, MmGT057 and MmGT060 we employed a “touchdown” cycling program consisting of: 95°C for 10 min, 30 cycles at 95°C for 30 s, annealing for 30 s, 118 and 72°C for 30 s; and a final step at 72°C for 10 min. The annealing step in the touchdown program decreased by 1°C every other cycle from 59°C until it reached 51°C; at that point, the remaining cycles continued at an annealing temperature of 51°C. Amplified fragments were separated through electrophoresis using Tris-Glycine buffer system on native polyacrylamide gels. We used 7% acrylamide-bisacrylamide 19:1 for AgGT19, AgGT29, AgGT90, MmGT046, MmGT054 and MmGT057 and 8% polyacrylamide gel for MmGT060. Gels were run at 280 V for 3 h and silver stained. Allele sizes were determined by comparison with a 10-bp DNA ladder molecular size standard (Invitrogen) (Additional file 3). The sex of adults was determined as indicated in [27], using the specific markers P2 and P8 for ZW sexual chromosomes. Only one adult from Marull was not sexed.

### Genetic analyses

Allelic richness, observed and expected heterozygosity, and tests for deviation from H-W equilibrium and linkage disequilibrium were performed with Arlequin 3.11 program [28], using default parameters and adult individuals only (N = 42). A random sample of 36 nestlings, taking one nestling from each chamber and a combined data set of the adults and nestlings (78 individuals) was also used to explore H-W equilibrium (data not shown). The presence of null alleles was investigated in the whole data set using the software Micro-Checker v2.2.3 [29] and ML-Relate [30]. The latter program was used to adjust allelic frequencies for null alleles.

We performed a molecular analysis of variance (AMOVA) [31] in Arlequin 3.11 [28] using adult individuals only, to estimate the contribution of hierarchical partitions to genetic variation (i.e., between localities, among individuals within localities and within individuals). We also estimated if fixation indices were significantly different from zero by means of 1,000 permutations.

We estimated patterns of sexual monogamy according to Gonçalves da Silva *et al.*[6]. The hypothesis of sexual monogamy predicts that both adult-juvenile and nestlings within chambers should have relatedness values close to 0.5 and the relationship among chambers should have values of 0 [32]. We used iRel program to evaluate the power of our markers for assessing dyads to a certain relationship category (i.e., full-sibs). This program computes expected misclassification rates as the fraction misclassified out of 1,000 simulated pairs of each category, using cut-off values [33]. The calculation of pairwise r was based on the method described in Li *et al.*[34], with each locus weighted using the method described in Lynch & Ritland [35] and Van de Casteele *et al.*[36] performed in Storm [37]. The test of significance was performed by comparing 95% confidence intervals around the mean.

We also used the program Storm [37] to calculate the mean relatedness (r) within chambers. This program provides a method to test the hypothesis that individuals within observed groups, in this case chambers, are more or less related than expected if the groups represented random associations of individuals with respect to relatedness. The calculation of r is based on the method described in Li *et al.*[34]. We performed 1,000 iterations of Monte Carlo simulations to generate expected distribution of average relatedness within a group. For each simulation, individuals were shuffled between groups keeping each group size constant. In addition, we estimated the average relatedness between chambers in those nests that had two or more chambers. To estimate 95% confidence intervals of mean value of chicks within chambers, 10,000 parametric bootstrap replications were performed. The estimations were carried out with boot package in R 2.12.2 [38].

ML-Relate software was used to estimate the relationship among nestlings; this program uses a maximum likelihood approach and accommodates for the occurrence of null alleles. Together with r values, the relationship estimated by ML-Relate was used to specify the kinship among nestlings from the same chamber or nest, when applicable.

Full-sibs and paternity and maternity of nestlings were identified by using the maximum likelihood method implemented in Colony 2.0.1.1 [39,40]. We used the full likelihood method option, the long option for the length of run and the allelic frequencies adjusted for null alleles. Longer runs are more likely to find the maximum likelihood configuration. According to the previous evidence, we selected the mating system option as “monogamous” and ran these parameters 10 times to find the best configuration. Finally, the possibility of parentage and sibship relationships from the other localities was excluded mutually.

Intraspecific brood parasitism was assumed in those broods with nestlings genetically unrelated with a) other nestlings in the clutch and, b) the genetic parents of the remaining nestlings (when data were available).

## Competing interests

The authors declare that they have no competing interests.

## Authors’ contributions

JJM collected the genetic data, analysed the data and contributed to the writing of the paper. MCdA helped collecting the genetic data, analysed the data and contributed to the writing of the paper. JFM designed the study, contributed to the field work and collection of samples, and reviewed several drafts and the final manuscript. EHB designed the study, collected samples in the field, performed research, and wrote the paper. All authors read and approved the final manuscript.

## Supplementary Material

Additional file 1 Monk parakeet nestlings and adults found in each chamber, inferred relationships and probable mating system. Click here for file

Additional file 2 **Genetic variation at the seven microsatellite loci in adult individuals of monk parakeet (*Myiopsitta monachus*).** Click here for file

Additional file 3 **Polyacrylamide gel electrophoresis patterns of microsatellite amplified in *M. monachus *using A) AgGT019, B) AgGT029, C) AgGT090, D) MmGT046, E) MmGT054, F) MmGT057, and G) MmGT060 markers.** M: 10 bp ladder marker (Invitrogen); M1: 100 bp ladder marker (Invitrogen). The chambers of provenance of each individual are indicated according to Figure 1. Click here for file
