# Supplementary material for: Genetic evidence of extra-pair paternity and intraspecific brood parasitism in the monk parakeet
Source: Front Zool. 2013 Nov 9;10:68. doi: 10.1186/1742-9994-10-68 (PMC3839639; doi:10.1186/1742-9994-10-68)
Supplement: Additional file 1 — Monk parakeet nestlings and adults found in each chamber, inferred relationships and probable mating system. [file 1742-9994-10-68-S1.docx]

**Additional file 1 Monk parakeet nestlings and adults found in each chamber, inferred relationships and probable mating system**

| **Locality** | **Nest** | **Chamber** | **Nestlings** | **Adults related to nestlings** | | **Adults found in chambers** | | **Breeding behavior** |
| --- | --- | --- | --- | --- | --- | --- | --- | --- |
|  |  |  |  | **Adult males** | **Adult females** | **Males** | **Females** |  |
| Marull | C1 | C11 | 3FS + 1HS | **F1xb** (3/4) |  | C117 |  | EPP |
| Marull | F1 | F11 | 6HS (3FS + 3FS) | **F1xb** (3/6) |  | **F1xb** | **F117** | EPP |
|  |  | F12 | 3FS + 1UR + 1UR |  | **F117** (1/5) | F1xa | F1xc | MON, IBP |
| Marull | G1 | G11 | 4FS | G116 (4/4) | G115 (4/4) | G116 | G115 | MON |
| Marull | H1 | H11 | 1 |  |  |  |  | UN |
| Marull | H2 | H21 | 4HS (2FS + 2FS) | H216 (3/4) |  | H216 | H215 | EPP |
| Marull | H3 | H31 | 5FS |  |  |  |  | MON |
| Marull | I1 | I11 | 3FS + 1HS |  |  |  |  | EPP |
| Marull | J1 | J11 | 6FS |  |  |  |  | MON |
| Marull | K1 | K11 | 4 (3FS + 1UR) | K115 (3/4) | K116 (3/4) | K115 | K116 | EPP, IBP |
|  |  | K12 | 1 |  |  | K125 | K121, K123, K124 | UN |
| Miramar | AB1 | AB11 | 3FS |  |  |  |  | MON |
| Miramar | L1 | L11 | 4FS |  |  |  |  | MON |
|  |  | L12 | 3FS + 1HS + 1HS |  |  |  | L121 | EPP |
| Miramar | L2 | L21 | 1 |  |  |  |  | UN |
|  |  | L22 | 5FS |  |  |  |  | MON |
|  |  | L23 | 6HS (3FS + 3FS) |  |  |  |  | EPP |
|  |  | L24 | 4FS + 1HS + 1HS |  |  |  |  | EPP |
| Miramar | M1 | M11 | 4FS | M116 (4/4) | M115 (4/4) | M116 | M115 | MON |
| Miramar | N1 | N11 | 2HS |  |  |  |  | EPP |
| Miramar | O1 | O11 | 2FS |  |  |  | O113 | MON |
| Miramar | Q1 | Q11 | 3FS |  |  | Q116 | Q1114 | MON |
| Miramar | R1 | R11 | 6FS | R118 (6/6) | R117 (6/6) | R118 | R117 | MON |
| Miramar | S1 | S11 | 2FS |  |  |  |  | MON |
|  |  | S12 | 3FS |  |  |  |  | MON |
| Miramar | T1 | T11 | 2HS | **T114** (1/2) |  | **T114**, T115 |  | EPP |
| Miramar | U1 | U11 | 4FS + 1HS |  | L2g1 (2/5) | **U117** | U116 | UN |
|  |  | U12 | 5HS (2FS + 3FS) | U126 (3/5) |  | U126 |  | EPP |
| Miramar | V1 | V11 | 4FS + 1HS + 1HS |  |  |  |  | EPP |
| Miramar | W1 | W11 | 5FS |  |  |  |  | MON |
| Miramar | W2 | W21 | 4FS | W215 (4/4) | W217 (4/4) | W215, W516 | W217 | MON |
| Miramar | W3 | W31 | 5HS (3FS + 2FS) | W317 (2/5) | W316 (2/5) | W317 | W316 | EPP |
|  |  | W32 | 2(1UR + 1UR) | **U117** (1/2) |  |  |  | MON, IBP |
| Miramar | W4 | W41 | 5HS (2FS + 2FS + 1HS) | **U117** (2/5)**, T114** (1/5**)** |  |  |  | EPP |
| Miramar | W5 | W51 | 6HS (4FS + 2FS) |  |  |  |  | EPP |
| Miramar | Y1 | Y11 | 5FS |  |  |  |  | MON |
| Miramar | Z1 | Z11 | 8FS |  | Z119 (8/8) | Z110 | Z119 | MON |

Nestlings: total number (siblings). FS: full-sibs, HS: half-sibs, UR: unrelated. Adults: Individual code (number of related/total number of nestlings in chamber). Rows shaded in grey: nests with more than one chamber. Breeding behaviour: MO= monogamy, EPP= extra-pair paternity, IBP= intra-brood parasitism, UN= uncertain. Special cases (in bold): F1xb: father of three FS nestlings in chamber C11 plus three FS nestlings in chamber F11, trapped in chamber F11. F117: mother of one unrelated nestling in chamber F12, trapped in same nest, chamber F11. T114: father of one HS nestling in chamber T11 plus one HS nestling in chamber W41, trapped in chamber U11. U117: father of one UR nestling in chamber W32 plus two FS in chamber W41, trapped in chamber U117.

**Comments**

We identified three cases of males having paternity relationship with nestlings in different nests, as follows: *F1xb:* father of 3 full siblings in nest C1 (single chamber), and father of three full-sibs in nest F1 (chamber 1). *U117*: father of one nestling (clutch of two) in nest W3 (chamber 2), and father of two full-sibs (clutch of five) in nest W4 (single chamber). It was trapped in nest U1*. T114*: father of one nestling (clutch of two) in nest T1 (single chamber), and father of two (clutch of five) in nest W4 (single chamber). It was trapped in nest T1.

Evidence for intra-brood parasitism was found in three chambers, involving four nestlings (3% of all nestlings sampled). Nest K1, single chamber: one unrelated nestling together with three full-sibs. The two genetic fathers of the three full-sibs were trapped in the same chamber. Nest F1, chamber 2 (of two): two unrelated individuals together with three full-sibs. The genetic mother of one of the unrelated nestlings was captured in the neighboring chamber. Nest W3, chamber #2 (of two): two unrelated nestlings only. The genetic father of one of the nestlings was captured in another nest within a 50-m range (U11).

We detected two cases with adult individuals in addition to male-female pairs, which are consistent with the presence of “trios” already reported by [8] [9]. Nest W2 (single chamber): clutch of 4 FS. Genetic father and mother captured in the chamber, plus an extra, unrelated male. Nest T1 (single chamber): clutch of 2 full-sibs. Two males found in the chamber, one of them is the genetic father of one of the nestlings.
