# Supplementary material for: Genetic evidence of extra-pair paternity and intraspecific brood parasitism in the monk parakeet
Source: Front Zool. 2013 Nov 9;10:68. doi: 10.1186/1742-9994-10-68 (PMC3839639; doi:10.1186/1742-9994-10-68)
Supplement: Additional file 2 — Genetic variation at the seven microsatellite loci in adult individuals of monk parakeet ( Myiopsitta monachus ). [file 1742-9994-10-68-S2.docx]

**Additional file 2 Genetic variation at the seven microsatellite loci in adult individuals of monk parakeet (*Myiopsitta monachus*)**

|  |  | *Marull* | *N*=16 |  |  | *Miramar* | *N*=26 |  |
| --- | --- | --- | --- | --- | --- | --- | --- | --- |
| **Locus** | **AR** | **He** | **Ho** | ***p*** | **AR** | **He** | **Ho** | ***p*** |
| AgGT019 | 10 | 0.90 | 0.69 | 0.13 | 14 | 0.92 | 0.80 | 0.01 |
| AgGT029 | 8 | 0.88 | 0.86 | 0.55 | 9 | 0.88 | 0.65 | 0.00 |
| AgGT090 | 5 | 0.69 | 0.56 | 0.02 | 7 | 0.71 | 0.38 | 0.00 |
| MmGT046 | 6 | 0.55 | 0.56 | 0.75 | 5 | 0.49 | 0.56 | 0.86 |
| MmGT054 | 9 | 0.77 | 0.13 | 0.00 | 11 | 0.63 | 0.33 | 0.00 |
| MmGT057 | 7 | 0.82 | 0.75 | 0.88 | 13 | 0.87 | 0.77 | 0.29 |
| MmGT060 | 4 | 0.62 | 0.63 | 0.21 | 6 | 0.65 | 0.58 | 0.29 |

*N*, numbers of individuals typed; AR, allelic richness, Ho, observed proportion of heterozygous individuals; He, expected proportion of heterozygous individuals; *P*, results of Hardy−Weinberg test.
