# Supplementary figures and images for: Genetic evidence of extra-pair paternity and intraspecific brood parasitism in the monk parakeet
Source: Front Zool. 2013 Nov 9;10:68. doi: 10.1186/1742-9994-10-68 (PMC3839639; doi:10.1186/1742-9994-10-68)

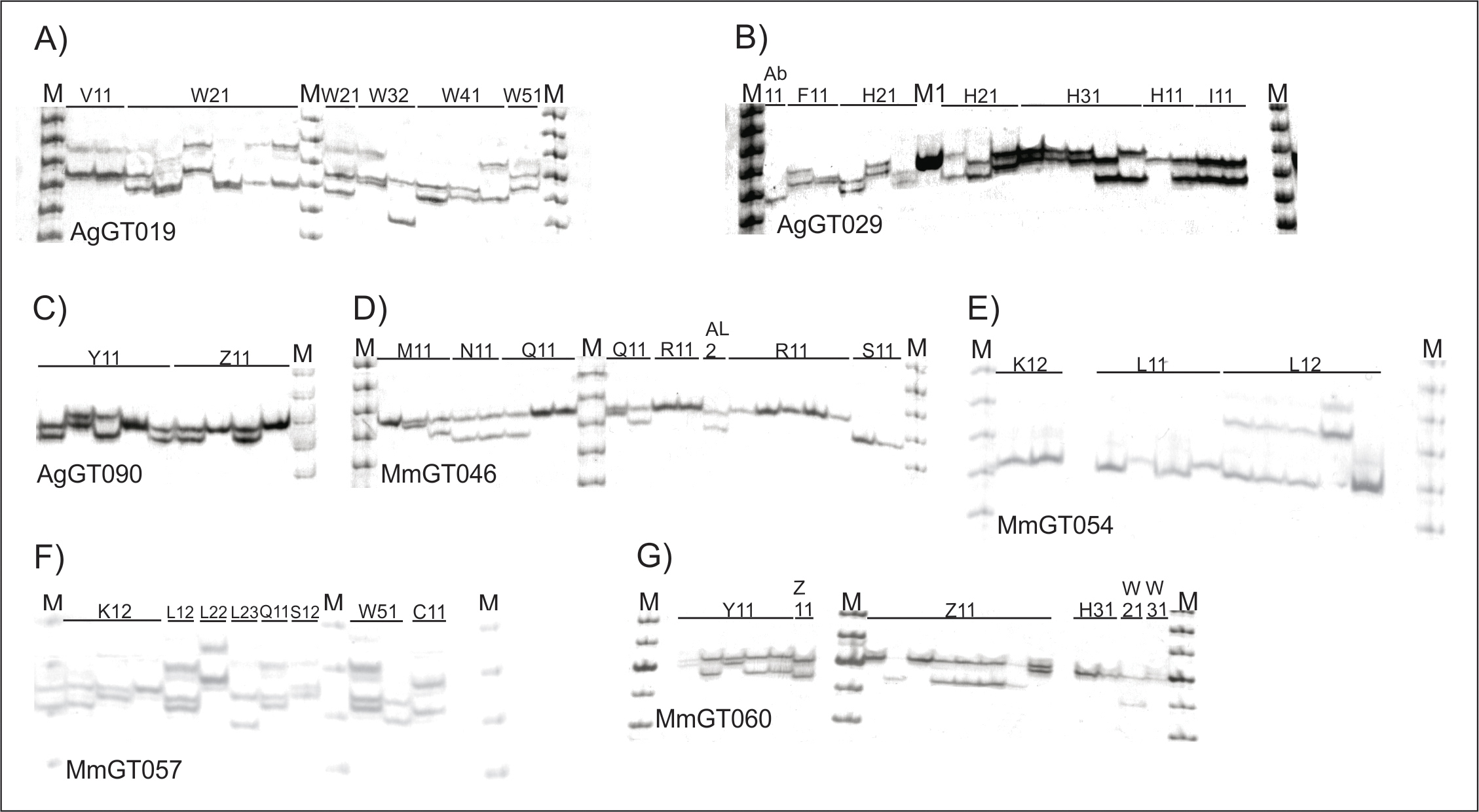

Supplement: Additional file 3 — Polyacrylamide gel electrophoresis patterns of microsatellite amplified in M. monachus using A) AgGT019, B) AgGT029, C) AgGT090, D) MmGT046, E) MmGT054, F) MmGT057, and G) MmGT060 markers. M: 10 bp ladder marker (Invitrogen); M1: 100 bp ladder marker (Invitrogen). The chambers of provenance of each individual are indicated according to Figure 1. [file 1742-9994-10-68-S3.jpeg]
